# Lexical Planning in Sentence Production Is Highly Incremental: Evidence from ERPs

**DOI:** 10.1371/journal.pone.0146359

**Published:** 2016-01-05

**Authors:** Li-Ming Zhao, Yu-Fang Yang

**Affiliations:** 1 Institute of Psychology, Chinese Academy of Sciences, Beijing, China; 2 Academy of Psychology and Behavior, Tianjin Normal University, Tianjin, China; Sun Yat-sen University, CHINA

## Abstract

The scope of lexical planning, which means how far ahead speakers plan lexically before they start producing an utterance, is an important issue for research into speech production, but remains highly controversial. The present research investigated this issue using the semantic blocking effect, which refers to the widely observed effects that participants take longer to say aloud the names of items in pictures when the pictures in a block of trials in an experiment depict items that belong to the same semantic category than different categories. As this effect is often interpreted as a reflection of difficulty in lexical selection, the current study took the semantic blocking effect and its associated pattern of event-related brain potentials (ERPs) as a proxy to test whether lexical planning during sentence production extends beyond the first noun when a subject noun-phrase includes two nouns, such as “The chair and the boat are both red” and “The chair above the boat is red”. The results showed a semantic blocking effect both in onset latencies and in ERPs during the utterance of the first noun of these complex noun-phrases but not for the second noun. The indication, therefore, is that the lexical planning scope does not encompass this second noun-phrase. Indeed, the present findings are in line with accounts that propose radically incremental lexical planning, in which speakers plan ahead only one word at a time. This study also provides a highly novel example of using ERPs to examine the production of long utterances, and it is hoped the present demonstration of the effectiveness of this approach inspires further application of ERP techniques in this area of research.

## Introduction

Linguistic planning refers to the necessary retrieval of words and syntactic building in preparation for producing fluent utterances. Most models of language production assume that the linguistic planning of an utterance is processed incrementally [1–3]. One implication is that utterance articulation can be initiated before all of the constituent words and the entirety of its structure are planned. Based on this incremental hypothesis, a long-standing issue concerns how far ahead speakers plan before they start producing an utterance. This issue is related to a number of important central questions in psycholinguistic studies of language production, such as the horizontal information flow (how different processing levels interact) in spoken sentence production [4–5].

Most psycholinguistic models of language production distinguished three major processing levels involved in conceptualizing, formulating, and ultimately articulating an utterance [6–7]. Additionally, Garrett [6] and Levelt [7] hypothesize that the formulating process consists of two distinct processing steps: grammatical encoding and phonological encoding, and lexical retrieval is considered as an important sub-component of grammatical encoding. Thus the issue of planning scope can be considered in the context of each of these different processing levels. However, the focus of the present study is primarily on the issue of lexical planning scope.

The lexical planning scope in speech production, that is how far ahead speakers plan lexically before they start producing an utterance, has been a particular focus of prior research in this area. It has primarily been investigated using classical paradigms for word production and, based on the findings from this research, various candidates for the scope of lexical planning have been proposed, including the first content word, functional phrase, or clause, among others.

Evidence that first noun phrase of an utterance forms the first unit of lexical planning scope in sentence production comes mostly from studies comparing the onset latencies of utterances with different structures that describe the relationship between two or more distinct objects. For example, Levelt and Maassen [8] asked participants to describe two moving objects on the screen, and found that onset latencies were longer for utterances that had a conjoined noun phrase as its subject (e.g., “The circle and the square move up”) than similar utterances that consisted of two conjoined sentences (e.g., “The circle moves up and the square moves up”). Using a similar task, Smith and Wheeldon [9] found longer onset latencies for sentences such as “The dog and the foot move above the kite” than sentences such as “The dog moves above the foot and the kite”. Existing evidence suggested that the phonological planning unit is limited to the first phonological word [10–12]. Consequently, the effects observed in these studies were unlikely to reflect this process. These researchers therefore claimed that conceptual and lexical planning scope were responsible for these effects, and that the planning scope for these component processes encompass the subject noun phrase rather than the whole sentence. However, this research was unable to establish if whole subject noun phrase or simply the head noun of the subject phrase served as the lexical planning scope, because in these studies only the head nouns were included in the subject noun phrase [9]. To clarify this question, Allum and Wheeldon [13] asked participants to describe two vertically presented pictures according to the color of the pictures, and compared the onset latencies for two types of sentences, one with a head noun modified by a prepositional phrase (PP) as the subject, such as “the dog above the flower is red” (henceforth “PP utterances”), and another with a conjoined noun phrase (CNP) as the subject, such as “the dog and the flower are both red” (henceforth “CNP utterances”). They found faster onset latencies for PP than CNP utterances, and took these findings to show that the conceptual and lexical planning scope encompass only the head nouns of the subject phrases.

Allum and Wheeldon [13] further tested the latency effects of CNP and PP utterances in Japanese, a head-final language, in which the modifier phrase of the PP utterances is produced before the head noun. Again, longer onset latencies were observed for the CNP utterances than for the PP utterances. As the initial phrase of PP utterances is no longer the head noun, they concluded that the conceptual and lexical planning scope is not the initial head noun or the subject noun phrase, and suggested that it instead encompasses a functional phrase. According to Allum and Wheeldon [13], the functional phrase represents a unit in the thematic representation of an utterance, but not necessarily the argument of the verb or the head of a verb argument phrase (p. 792). The subject noun phrase in a CNP utterance serves as the agent, while the subject noun-phrase of a PP utterance consists of two smaller functional phrases. Thus, the longer onset latencies for the CNP utterances could be attributed to the fact that its first functional phrase is longer.

This functional phrase hypothesis provides a novel definition of the lexical planning unit, but calls for further investigation of this issue. First, as utterance articulation can be initiated before all of the constituent words and the entirety of its structure are planned, the onset latency will include the time required for conceptual, lexical, and structural planning. Thus the evidence above coming from the latency effects of different utterance formats may not discriminate among these different planning scopes. Moreover, Wheeldon and colleagues [14] have suggested that the scope of lexical and structural planning are not always coincident, and that the latency effect of the initial phrase size may be attributed to structural planning only. However, some studies tried to solve this problem for the functional phrase hypothesis. For example, Zhao, Alario, and Yang [15] used a similar task to Allum and Wheeldon (2007) but asked participants to preview the pictures without a color cue before each stimulus was presented, which means that participants could retrieve both the names of the pictures but did not know which sentence structure would be used during the previewing period. The time required for the conceptual and lexical planning should therefore no longer influence onset latencies, and the latency effect between CNP and PP utterances was indeed absent in this preview condition. Thus the researchers confirmed that the latency effect previously observed should be attributed to conceptual and lexical planning rather than structural planning, but whether it is conceptual or lexical planning that drives the effect remains unclear. Allum and Wheeldon [16] also used a previewing task, in which the previewing effect for each sentence type was obtained by comparing performance when the bottom picture (the second noun) was previewed or not. The facilitation effect of previewing the bottom picture was found for CNP utterances only, suggesting that the second noun was planned in the CNP but not in the PP utterances. Nevertheless, it remains unclear whether this preview effect facilitated conceptual or lexical processing of the second noun in CNP but not PP utterances.

Another approach to testing lexical planning scope takes semantic context effects in lexical production as an index. Various candidates other than the first noun phrase have been suggested by this line of research [10, 17–18]. For instance, Griffin [10] asked participants to produce sentence with the structure “the N1 and the N2 are above the N3” in response to three pictured objects while manipulating the codability (the number of alternative names, such as *ship*, *boat*, or *sailboat* for a schooner) of each picture (N1, N2, and N3). Onset latencies were affected by the codability of the N1, but not N2 or N3. As codability influences lexical selection, it was concluded that speakers often incrementally select nouns (one by one) in fluent utterances. This claim is called the radically incremental hypothesis. With the similar logic, Zhao and Yang [19] tested the functional phrase hypothesis using a picture-word interference paradigm. In this task, two pictures were presented vertically with a distractor embedded in one of the pictures. Participants were asked to produce a sentence with the picture names like CNP and PP utterances while trying to ignore the distractor. A semantic interference effect was observed only for the first noun of both CNP and PP utterances. That is, onset latencies were longer when the distractor was semantically related (category coordinate) to the first noun than when they were unrelated. As the semantic interference effect is often interpreted as a reflection of difficulty in lexical selection during word production [20] (but see [21]), the results of Zhao and Yang [19] are generally taken to support the radically incremental hypothesis rather than the functional phrase hypothesis.

But to reconcile discrepancies in findings and conclusions, some researchers have suggested that the lexical planning scope may be flexible rather than fixed. The reason for this is that a number of factors could modulate the planning patterns, such as time pressure [22], cognitive load and variable utterance formats [23], and the availability of the words and syntactic structures [4, 14]. However, according to the previous evidence, a certain planning unit may be preferred in certain speaking situation or conditions, even though the lexical planning scope is flexible. Moreover, an appropriate planning scope is critical for speech fluency. The current study aimed to test whether the functional phrase could be a preferred unit of lexical planning as expected according to the conditions in the circumstance, using the semantic blocking effect as an index of lexical selection and with EEG recordings.

The semantic blocking effect is a stable effect of semantic context in the blocked-cyclic naming paradigm, in which each block consists of several repeated cycles of a small set of pictures. In the semantically homogeneous condition, all the items in the block represent the same semantic category (e.g. animals), and in the semantically heterogeneous (or mixed) condition each item is from a different semantic category. It has been observed that in the picture naming task the naming latencies in the homogeneous condition are significantly longer than in the heterogeneous condition [24–25]. This latency effect, named the semantic blocking effect, is interpreted as an effect of lexical selection by some researchers [26–27] (but see [28]), and frequently used to investigate the time-course and neuro-anatomical regions of word selection [29–31]. Indeed, semantic blocking is associated with an ERP component around 200ms post picture onset [30, 32].

### Current experiments

In the current study the semantic blocking effect was used to test the lexical planning scope. Two experiments are reported that examined effects of semantic blocking for CNP and PP utterances consisting of two nouns. The purpose of Experiment 1 was to test whether the semantic blocking effect was stable in sentence production. In this experiment, the first noun of each utterance was manipulated to be either homogeneous or heterogeneous in each block of trials, while the second noun was always heterogeneous in all blocks. As the semantic blocking effect is widely observed in the picture naming experiments, and the first noun of each sentence is considered to be completely retrieved before speech onset, it was expected that a semantic blocking effect would be observed in speech onset latencies and corresponding ERP components. If so, the semantic blocking effect would be considered to be a stable effect during sentence production, and therefore could be used to test lexical planning scope.

The aim of Experiment 2 was to test whether the second noun was lexically planned before speech onset. In this experiment, the second noun of the utterances was manipulated so that is was semantically homogeneous or heterogeneous in each block of trials, while the first noun was heterogeneous in all blocks. According to the functional phrase hypothesis, a semantic blocking effect in speech onset latencies should be observed for CNP utterances, but not PPs. By comparisons, as the radically incremental hypothesis would predict no semantic blocking effect for either CNP or PP utterances; while if the lexical planning encompasses the whole subject noun phrase, there would be semantic blocking effect for both CNP and PP utterances.

So far neurophysiological technology has been used broadly in word naming tasks in which participants name a single object in a picture, but quite little in sentence production. One of the main problems is that ERP data are very sensitive to the physical movement that takes place during speech production. Thus ERP data are only valid in the planning period, which limits the questions concerning speech production that can be studied effectively using ERPs. However, this was not a major issue for the present research because we were principally interested in the processes that take place during lexical planning, prior to the onset of speech. ERP data can provide insights into the neural basis of this lexical planning. Additionally, findings by Janssen et al. [32] had suggested that ERPs may show a different pattern from that observed in behavioral, and so we considered it important to study ERPs in addition to behavioral data to gain a fuller understanding of the nature of lexical processing. Finally, because ERPs provide a fine-grained account of activity that takes place over time, they have the potential to enable us to differentiate components that are associated with lexical planning and structural planning. Indeed, when a long utterance is going to be produced, there are many processes involved in the planning period, including lexical and structural planning, and so the ERP record will reflect the operation of these various processes. Therefore, to separate effects of structural planning and lexical planning in the present experiment, each block used only one sentence structure, CNP or PP. This repetition of sentence structure was intentional to reduce effects of structure planning. This approach was quite different from that adopted in previous studies of the functional phrase hypothesis [8, 15–16], in which sentence structures were deliberately varied. Moreover, according to the classic design of semantic blocking effect, each picture is repeated several times in each block. This was also different from the approach of previous studies, in which each picture appeared once in each block. The deliberate repetitions of structures and lexical items in the present study might well encourage speakers to adopt larger lexical planning scope [4, 14]. Thus it would not be surprising if we observed that the functional phrase or even larger unit was the lexical planning scope. However, if there was no semantic blocking effect on the second noun of both CNP and PP utterances, that will provide strong evidence for the claims of radically incremental hypothesis [10], and against the claim that the functional phrase as the preferred lexical planning unit.

## Experiment 1

As mentioned in the Introduction, the semantic blocking effect in onset latencies of single picture naming is consistently observed in the literature, and interpreted as a reflection of lexical selection. It is reasonable to use this effect as a proxy to test the lexical planning scope. But before that we need to know whether the semantic blocking effect can be extended to a sentence production task. The aim of this experiment was to clarify this issue.

As the first noun of each sentence is considered to be completely retrieved before speech onset, semantic blocking effect in the RTs (for speech onset) and ERP components would be expected when comparing the homogeneous and heterogeneous conditions for the first noun. To reduce the influence of structural effects, the sentence structure was kept unvaried in each block. In addition, to ensure there was no semantic effect due to the retrieval of the second noun, this was presented in heterogeneous conditions in all blocks.

### Method

#### Participants

Twenty-four undergraduate and graduate students participated in the experiment. They were all native Chinese speakers with normal or corrected-to-normal vision, and were paid for their participation. This study was approved by the Institutional Review Board of the Institute of Psychology at the Chinese Academy of Science. All participants provided written informed consents.

#### Materials

Eighteen pictures were selected from the picture pool of Snodgrass and Vanderwart [33]. They were divided into 6 semantic categories, each consisting of 3 items (see S1 Appendix). Three categories (zoo animals, fruits, and furniture) served as the first noun in the produced utterances, and were presented always at the top position (called top groups); while the other three categories (transports, musical instruments, and body parts) were used as the second noun, and always presented at the bottom position (called the bottom groups). For the top group, items from the same category were combined to form three homogeneous sets; while items from three categories respectively were combined to form three heterogeneous sets. For the bottom group, items from different categories were combined to form three heterogeneous sets, and then recombined to form another three heterogeneous sets. Each of the six heterogeneous sets for bottom groups was paired with one of the top group to form six blocks (see S1 Appendix for a detailed arrangement). Each block was produced in CNP and PP utterances respectively, forming 12 blocks totally. In the blocks of CNP utterances, both pictures in each trial were presented in red lines; while in the blocks of PP utterances, the top picture was in white lines and the bottom picture was in red lines. Six filler items were presented for practice, and they were not from the experimental categories. There were two practice blocks, one in CNP utterances and the other in PPs.

#### Design

Semantic context for the top groups (homogeneous vs. heterogeneous) and sentence type (CNP vs. PP) were manipulated as within-participant factors. There were twelve experimental blocks (three homogeneous blocks in CNP, and three in PP; three heterogeneous blocks in CNP, and three in PP). The homogeneous and heterogeneous blocks were presented in alternative orders, and the CNP and PP blocks were presented in an AABB design. For the first three trials in each block, each of the six target pictures (three pictures from the top group paired with three pictures from the bottom group) was presented once. For the rest trials in the block, each of the target pictures was presented six times, each top item paired with each bottom item twice, in a pseudorandom order, so that the same picture never appeared in consecutive trials.

#### Procedure

Participants were seated in front of the computer screen, at a distance of about 60 cm, and tested individually. A fixation point appeared on the screen for 1,000 ms. Then the pair of pictures appeared for 4,000 ms. Participants were asked to name pictures with the required syntactic structures as accurately and quickly as possible. There was a blank interval of 2,000 ms between consecutive trials. Before starting each block, the six pictures used in this block were presented successively in a random order companied with their names, for participants to get familiar with them. Participants were asked to produce one sentence type (CNP or PP) through the whole block. The screen’s background remained black. The whole testing session lasted about 40 min, and participants had a short break between blocks.

#### ERP recording and analyses

The continuous EEG was recorded using an elastic cap equipped with 64 Ag/AgCl electrodes according to the International 10–20 system. The EEG data was amplified via AC amplifiers, at a sampling rate of 500 Hz (band pass filter = 0.05 Hz–100 Hz). The right mastoid electrode served as the online reference. An electrode placed between Fz and Cz served as the ground. The horizontal EOG was measured by placing two electrodes at the outer canthi, and the vertical EOG was measured with two electrodes placed above and below the left eye. All electrode impedances were kept below 5 KΩ during the experiment.

NeuroScan software 4.3 was used to preprocess the raw ERP data. Ocular artifacts were corrected by NeuroScan software [34]. The data were offline re-referenced using the algebraic average of two mastoids, and passed through 0.1–30Hz band (24dB/oct) filters. The data were segmented from 100ms before to 600ms after the stimulus onset (the onset of the picture pairs). A baseline correction was applied from 100 to 0 ms preceding stimulus onset. The artifact rejection criterion was ±100μV. The first occurrence of each picture (the first three trials) in each block was not included in further analyses. Finally, we excluded the ERP data of the trials on which the participants made production errors (see the types in details in the behavioral analysis), or the speech onset latencies were less than 600 ms. On average, 17% of all trials were rejected. Trials were then averaged in each condition for each participant, and this average was used for further statistical analysis. For the CNP utterances, there were 1121 out of 1296 (86.5%) trials in the homogeneous condition, and 1044 out of 1296 (80.6%) trials in the heterogeneous condition. For the PP utterances, there were 1075 out of 1296 (82.9%) trials in the homogeneous condition, and 1047 out of 1296 (80.8%) in the heterogeneous condition.

### Results

#### Behavioral results

**Fig 1:**
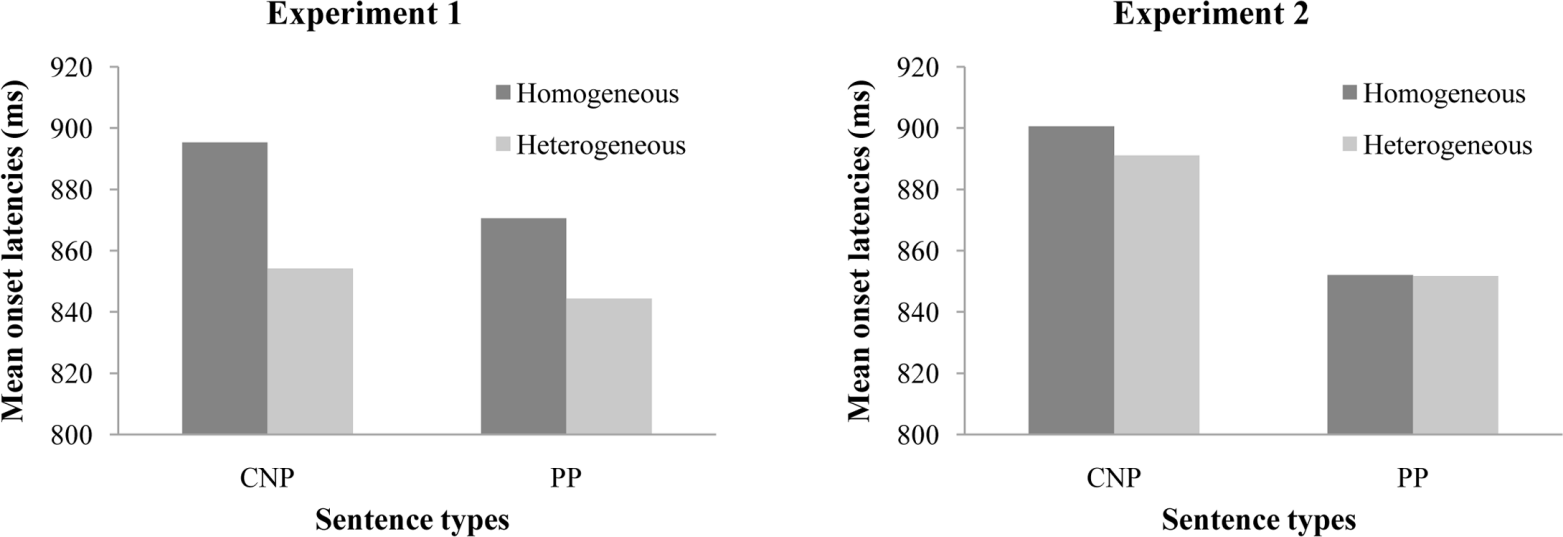
The mean onset latencies in each condition in Experiments 1 and 2 (Session 1).

Three types of responses were scored as production errors: (1) using unexpected content words, including picture names and the adjectives; (2) using incorrect syntax; and (3) fluency problems (repairing, stuttering, hesitation, and production of nonverbal sounds that triggered the voice key). Outliers were defined as latencies less than 300ms, more than 3000ms, or exceeding more than three standard deviations from a participant's mean latency. Latencies on error trials, outliers, and recording failures amounted to 4% of the data and were excluded from the latency analyses. As the semantic blocking effect is targeted, the first occurrence of each picture (the first three trials) in each block was also excluded. The mean onset latencies in each condition were shown in Fig 1.

Two separate analyses were carried out with participants (*F*_1_ / *t*_1_) and items (*F*_2_ / *t*_2_) as random factors. The factor of sentence type (CNP vs. PP) was analyzed within participants and between items. The factor of semantic context (homogeneous vs. heterogeneous) was analyzed within participants and items.

In the analyses of onset latencies, the main effect of semantic context was significant (*F*_1_(1,23) = 7.30, MSE = 3,461, *p* < .05; *F*_2_(1,16) = 14.07, MSE = 644, *p* < .01). The main effect of sentence type was significant for participants, but not for items (*F*_1_(1,23) = 6.40, MSE = 1,246, *p* < .05; *F*_2_(1,16) = 1.17, MSE = 2,832, *p* = .3). There was no interaction between semantic context and sentence type (*F*_1_(1,23) = 2.10, MSE = 677, *p* = .16; *F*_2_ < 1). The error rates were low (1% on average) and not analyzed further.

#### Electrophysiological results

For ERP results, the overall repeated measures ANOVAs were conducted on mean amplitudes of consecutive 50 ms time windows, from the onset of the picture pair to 600 ms after picture onset. This analysis included Sentence Type (CNP vs. PP) and Semantic Context (homogeneous vs. heterogeneous) as within-participant factors, and other two factors relevant to clusters of electrodes. That is, Anteriority with three levels (anterior, central, and posterior) and Hemisphere with two levels (left and right). As a result, six regions were considered: Anterior-Left (FP1, AF3, F1, F3, F5, F7), Central-Left (FC5, FC3, C5, C3, CP5, CP3), Posterior-Left (P5, P3, P1, PO5, PO3, O1), Anterior-Right (FP2, AF4, F2, F4, F6, F8), Central-Right (FC4, FC6, C4, C6, CP4, CP6), and Posterior-Right (P2, P4, P6, PO4, PO6, O2). The results were summarized in Table 1.

**Table 1:** Time Course of the Main ERP Effects in Experiment 1 with scalp distribution in brackets.

Time Window (ms) Post-target onset	Sentence Type Effect	Semantic Blocking Effect
0–50		
50–100	*F*(1, 23) = 6.42* (Central-Right)	
	*F*(1, 23) = 5.07* (Posterior-Right)	
100–150	*F*(1, 23) = 10.63** (Anterior)	
	*F*(1, 23) = 14.95** (Central)	
	*F* = 7.01* (Posterior-Left)	
150–200	*F*(1, 23) = 4.33* (Posterior)	
200–250	*F*(1, 23) = 10.14** (Anterior)	
250–300		*F*(1, 23) = 6.95* (Central)
300–350	*F*(1, 23) = 6.71* (Anterior)	*F*(1, 23) = 5.59* (Anterior)
		*F*(1, 23) = 4.73* (Central)
350–400	*F*(1, 23) = 10.20** (Anterior)	
	*F*(1, 23) = 6.43* (Central)	

The reported *F* values are the results of ANOVAs with Sentence Type (two levels) and Semantic Context (two levels) of the areas in brackets (Anteriority with three levels, and Hemisphere with two levels. Non-specific regions in brackets means the *F* values are across these regions. For example, “Anterior” in brackets means that the *F* value is across the two levels of Hemisphere. If the *F* value is not followed by brackets, it means that this *F* value is across all the six scalp areas). Only significant effects were presented. There was no significant effect from 400ms to 600 ms post target onset, thus were not presented.

*p < .05

**p < .01.

**Fig 2:**
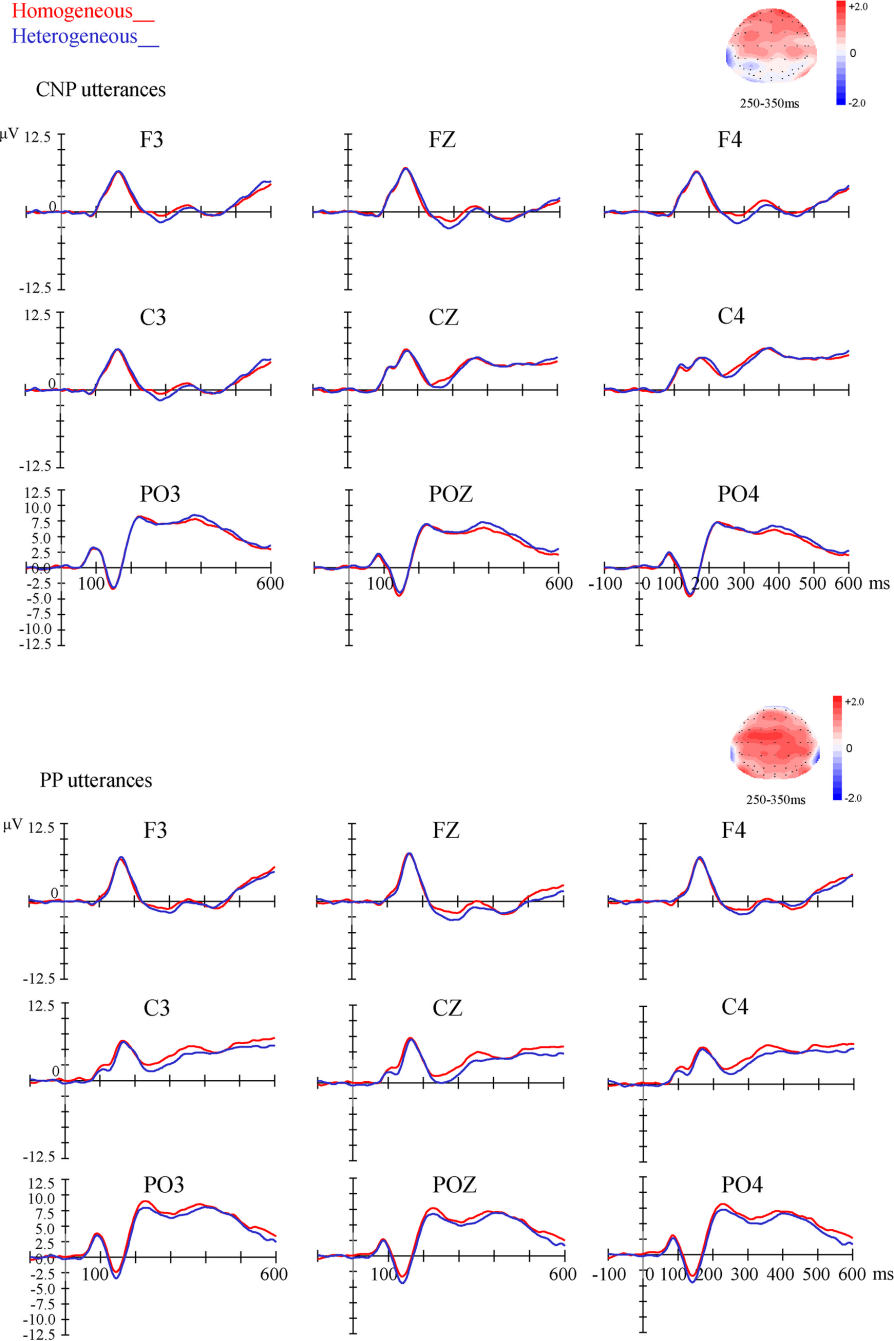
Semantic blocking effect of ERPs. Average ERPs from 100 ms before up to 600 ms after the onset of the picture pair, for homogeneous and heterogeneous blocks in CNP and PP utterances respectively, at the 9 electrodes as an example in each area (anterior, central, and posterior multiply with left, central, and right). Positivity is plotted upwards to compare with the results of Janssen et al. [32]. The scalp distribution of the semantic blocking effect (homogeneous condition *minus* heterogeneous condition) is presented in the time window of 250–350ms.

**Fig 3:**
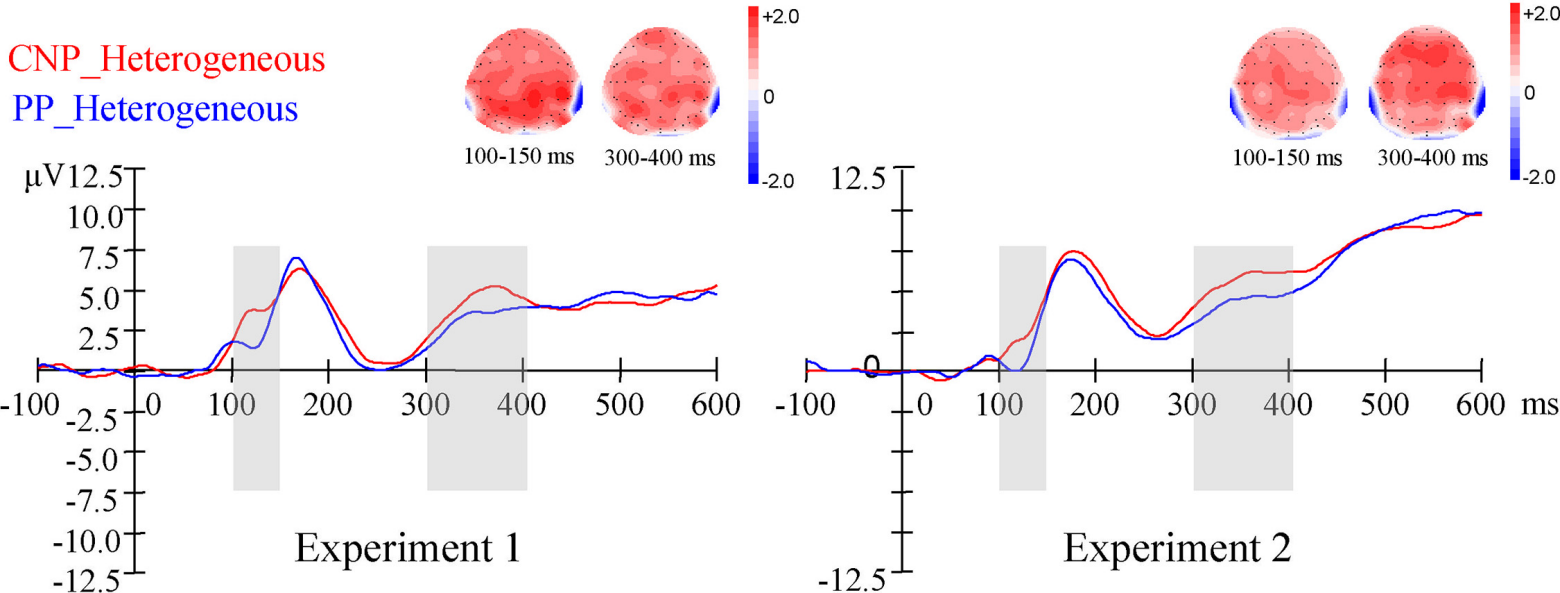
Sentence type effect of ERPs. Average ERPs from 100 ms before up to 600 ms after the onset of the picture pair, for CNP utterances in heterogeneous blocks and PP utterances in heterogeneous blocks, at the CZ electrode as an example. Positivity is plotted upwards. The scalp distribution of the sentence type effect (CNP *minus* PP) is presented in the time window of 100–150ms and 300–400ms.

There was a main effect of Semantic Context in the window of 250–350 ms, with a larger negativity in the heterogeneous condition compared to the homogeneous condition, and with an anterior and central distribution (see Fig 2). The ERP effect of Sentence Type was presented in Fig 3, taking the CZ electrode for instance.

### Discussion

Experiment 1 tested whether the semantic blocking effect in RTs and ERPs observed in single word production could be extended to overt sentence production.

Firstly, a semantic blocking effect in onset latencies was observed for both CNP and PP utterances as expected. Secondly, the semantic blocking effect in ERPs was also observed for both CNP and PP utterances. The ERP component distributed at anterior and central electrodes, and in the time window of 250–350 ms, which is consistent with previous results [29, 32]. The result showed that the semantic blocking effect in onset latencies and ERPs observed in single word production can be extended to overt sentence production, although the utterances are much more complicated and the syntactic processing for sentence structures are involved in the speech planning. Moreover, the semantic blocking effect of ERPs showed a larger negative wave for the heterogeneous condition compared to the homogeneous condition, which is in line with the findings of Janssen et al. [32], but different from Aristei et al. [29] in the polarity of the semantic blocking effect.

The effect of sentence type was observed in onset latencies, and the ERP component distributed at anterior and central electrodes, pronounced in the time window of 100–150ms and 300–400ms. From visual inspection, the ERPs in the time window of 100–150ms revealed a P1-N1 complex that is typically associated with the presentation of visual stimuli. This may result from the different visual displays (cued by color) of these two types of utterances. It might be that the ERPs in the time window of 300–400ms reflected the syntactic processing of the sentence structures. However, the lexical planning scope was not tested yet in this experiment. According to the functional phrase hypothesis, the second noun is lexically selected before speech onset for the CNP utterances, but not for the PP utterances. Thus the ERP effect of Sentence Type in the time window of 300–400ms may also reflect the retrieval of the second noun. This is tested in the Experiment 2.

## Experiment 2

The goal of Experiment 2 was to test whether the second noun in CNP and PP utterances was lexically selected before speech onset, that is, to test the functional phrase hypothesis on the lexical planning scope. If the second noun is lexically retrieved before speech onset for certain sentence types, the semantic blocking effect as in Experiment 1 should be observed for this type of sentences. To be specific, according to the functional phrase hypothesis, there would be semantic blocking effect in onset latencies and ERPs only for CNP utterances, similar to the one observed in Experiment 1. However, according to the radically incremental hypothesis, a semantic blocking effect in onset latencies should not be observed for either CNP or PP utterances. Moreover, if lexical planning encompasses the whole subject noun phrase, there should be a semantic blocking effect for both CNP and PP utterances.

So far the semantic blocking effect in ERPs was expected to coincide with that in onset latencies [29–30]. However, according to the interpretation of Janssen et al. [32], the ERP effect of semantic blocking reflects input processes rather than the lexical selection of the output processes. In light of this assumption the ERP component of any semantic blocking effect would not coincide necessarily with the semantic blocking effect in onset latencies. To be specific, if the corresponding ERP effect reflects input processes, and the latency difference between CNP and PP utterances results from input processes for the second noun, a semantic blocking effect in ERPs would be observed for CNP utterances only, even though the semantic blocking effect in onset latencies is absent for both CNP and PP utterances.

### Method

#### Participants

Twenty-four undergraduate and graduate students participated in the experiment. None of them had participated in Experiment 1. They were all native Chinese speakers with normal or corrected-to-normal vision, and were paid for their participation. All participants provided written informed consent.

#### Design

In Experiment 2, the materials and procedure were the same as in Experiment 1, except that the Semantic Context was manipulated for the second noun (bottom picture) instead of the first one (top picture). The experiment consisted of two sessions. In the first session, all the trials were exactly the same as in Experiment 1, except that the positions of the two pictures in each stimulus pair were exchanged. As a result, the semantic context (homogeneous vs. heterogeneous) was manipulated for the bottom picture, and the critical items were all from the three categories (zoo animals, fruits, and furniture) as in Experiment 1 (see S2 Appendix for details). This manipulation made this session (Session 1) comparable to Experiment 1 from the perspective of its items. After completion of this first session, and a short break, participants took part in a second session. This session was also similar to the Experiment 1, except that items from the bottom group were combined to form homogeneous and heterogeneous sets while the items from the top group were combined to form heterogeneous sets only. As a result, the critical items in this session (Session 2) came from the other three categories (transports, musical instruments, and body parts) as shown in S2 Appendix. The picture color was set to induce CNP and PP utterances, the same as in Experiment 1. The whole testing session lasted about 80 min, and breaks between blocks were allowed.

### Results

#### Behavioral results

With the same exclusion criteria as in Experiment 1, 3.4% of the data were removed from the latency analysis. The first occurrence of each picture (the first three trials) in each block was also excluded as in Experiment 1. The mean onset latencies in each condition for Session 1 were shown in Fig 1, and the same pattern was observed in Session 2. The latencies in the heterogeneous condition in Experiment 2 are as long as in the homogeneous condition in Experiment 1. This may be because the first nouns of the utterances in these two experiments are different, and according to our findings only the first noun of the utterance is lexically planned before speech onset.

The same analyses as in Experiment 1 were conducted here for two sessions of the Experiment 2 separately.

**Session 1:** In the analyses of onset latencies, the main effect of Sentence Type was significant (*F*_1_(1,23) = 20.21, MSE = 2,228, *p* < .001; *F*_2_(1,16) = 38.71, MSE = 437, *p* < .001). There was no other effect and interactions reached significance (*F*s < 1). The error rates were low (1% on average), and not analyzed further.

**Session 2:** In the analyses of onset latencies, same pattern was observed as Session 1. Only the main effect of Sentence Type was significant (*F*_1_(1,23) = 8.26, MSE = 3,885, *p* < .01; *F*_2_(1,16) = 24.23, MSE = 477, *p* < .001). There was no other effect and interactions (*F*s < 1, except for the interaction: *F*_1_(1,23) = 1.46, MSE = 563, *p* = .24). The error rates were low (1% on average) and thus were not analyzed further.

#### Electrophysiological results

The same analyses as in Experiment 1 were conducted here for two sessions of the Experiment 2. The results of the overall ANOVA with scalp distribution are summarized in Table 2 and Table 3, for Session 1 and Session 2 separately (Only significant effects were presented).

**Table 2:** Time Course of the Main ERP Effects in Experiment 2_Session 1 with scalp distribution in brackets.

Time Window (ms) Post-target onset	Sentence Type Effect	Semantic Blocking Effect
0–50		
50–100		
100–150	*F*(1, 23) = 9.38**	
150–200		
200–250		
250–300		
300–350	*F*(1, 23) = 7.04* (Central)	
350–400	*F*(1, 23) = 6.24* (Central)	

The analysis and the presentation were similar to the Table 1.

*p < .05

**p < .01.

**Table 3:** Time Course of the Main ERP Effects in Experiment 2_Session 2 with scalp distribution in brackets.

Time Window (ms) Post-target onset	Sentence Type Effect	Semantic Blocking Effect
0–50		
50–100	*F*(1, 23) = 8.70** (Left)	
100–150	*F*(1, 23) = 4.35** (Anterior)	
	*F*(1, 23) = 5.87** (Central)	
150–200	*F*(1, 23) = 4.98** (Posterior)	
200–250	*F*(1, 23) = 4.73** (Anterior)	
	*F*(1, 23) = 4.87** (Central)	
250–300		
300–350	*F*(1, 23) = 4.81** (Central-Right)	
350–400	*F*(1, 23) = 4.28** (Anterior-Right)	
	*F*(1, 23) = 6.06** (Central)	
400–450	*F*(1, 23) = 5.37** (Anterior)	
	*F*(1, 23) = 4.77** (Central)	

The analysis and the presentation were similar to the Table 1. There was no significant effect from 450ms to 600 ms post target onset, thus were not presented.

**p < .01.

**Session 1:** The analysis of the first session revealed no significant effect of Semantic Context. There was a main effect of Sentence Type in the window of 300–400 ms with a central distribution, and in an isolated time window 100–150 ms across whole brain scalp, with a larger negativity for the PP utterances compared to the CNP utterances (see Fig 3).

**Session 2:** The analysis of the second session showed similar pattern as Session 1 and revealed no significant effect of Semantic Context. The main effect of Sentence Type was shown in the time window of 300–450 ms and in other two isolated time windows (100–150 ms and 200–250 ms) with a larger negativity for the PP utterances compared to the CNP utterances, with an anterior and central distribution.

### Discussion

The second noun in CNP and PP utterances was manipulated in homogeneous and heterogeneous conditions to test whether it is lexically selected before speech onset. To confirm the results, there were two sessions with the same design as Experiment 1, but with rearrangement of the items. It is quite clear that the semantic blocking effect in onset latencies and ERPs observed in Experiment 1 was absent for both sessions and both sentence types in Experiment 2. These results showed no sign that the second noun of CNP and PP utterances, no matter what syntactic role it plays, is lexically selected before speech onset. Even if the ERP effect of semantic blocking reflects input processes rather than the lexical selection of the output processes, the absence of this ERP effect would indicate that the second noun has not been processed in a much earlier stage, that is, the input processes.

An effect of sentence type was observed in both Experiments 1 and 2. Firstly, the production of CNP utterances requires more time to prepare than the PP utterances. This is consistent with the functional phrase hypothesis [13, 15]. However, as the semantic blocking effect on the first and second nouns did not show any difference between these two sentence types, suggesting the similar lexical planning scope for CNP and PP utterances, the sentence structure effect in onset latencies must result from factors other than lexical planning, such as input processes or syntactic processing. Secondly, the effect of sentence type in ERPs, with larger negativity for the PP utterances compared to the CNP utterances, was observed consistently at anterior and central electrodes in the time window of 100–150ms and 300–400ms. According to our previous discussion, the ERP effect in the time window of 100–150ms revealed a P1-N1 complex that is associated with the presentation of visual stimuli, and the effect in the time window of 300–400ms likely reflected the syntactic processing of the sentence structures. Thus considering the semantic blocking effect in 2507#x2013;350ms time window at the same time, these results suggest that the first content word is processed slightly prior to the processing of syntactic structures, which is in line with the incremental production model [35] (suggesting that the conceptually activated words tend to be produced early and influence the processing of syntactic structure). However, this speculation needs further examination, as the effect in the time window of 300–400ms may reflect other processes related to the visual factors, such as semantic categorization [36].

## General Discussion

The lexical planning scope in speech production is an important issue in psycholinguistics, but highly controversial. The first functional phrase, among others, has been proposed as the candidates for lexical planning scope in sentence production, which is called the functional phrase hypothesis. This hypothesis provides a novel definition of the lexical planning unit, but calls for further investigation of this issue.

To test the functional phrase hypothesis, two semantic blocking experiments were conducted with ERP recordings. In Experiment 1, the first noun of the utterances was manipulated in homogeneous and heterogeneous conditions, while the second noun was always presented in a heterogeneous condition. A semantic blocking effect in onset latencies and ERPs were observed for both CNP and PP utterances, similar to the effect shown in previous research in a single-word picture-naming task. It indicates that the semantic blocking effect is also obtained in a sentence production task, and so can be used to investigate the scope of lexical planning during speech production. In Experiment 2, the manipulation of semantic context was focused on the second noun. That is, the second noun was either in homogeneous or heterogeneous conditions, while the first noun was always in a heterogeneous condition. With this manipulation no semantic blocking effect was found both in onset latencies and ERPs. The results of this study therefore suggest that speakers lexically planned only for the first noun before speech onset, which is consistent with the prediction of radically incremental hypothesis [10]. That is, only the first noun is selected before speech onset.

The present results were more consistent with the first noun as the lexical planning scope [10] than the first noun phrase [8–9, 13, 15–16] or clause [37]. The evidence in favor of the phrasal planning scope is mainly based on the results of comparing onset latencies across utterance formats, in the task of verbal descriptions of two or more distinct objects. However, the onset latencies obtained in these cases may include the time span for conceptual, lexical, and structural planning, and it is difficult to determine which of these is responsible for the latency effect. Wheeldon and colleagues [14] claimed that the scope of lexical and structural planning need not always coincide. Thus it is difficult in attributing latency effects across utterance formats observed in evidence supporting the phrasal hypothesis to lexical access instead of syntactic planning. Thus it is easy to understand why the results of current study are consistent with Griffin’s [10] and Zhao and Yang’s [19] conclusions. Griffin [10] manipulated the codability of nouns to test the lexical planning scope for its potential influence on the lexical selection, and therefore avoided across-utterance comparisons and concluded on the comparison (i.e., codability effect) for the same sentence structure. Similarly in Zhao and Yang’s [19] picture-word interference study, the semantic interference effect was tested by comparing semantically related and unrelated conditions for the same utterances.

However, with the picture-word interference paradigm and within-utterance comparisons, Meyer [37] obtained contradictory evidence. Her results showed a semantic interference effect in the onset latencies for the second noun of phrases (e.g., “the dog and the flower”) and sentences (e.g., “The dog is next to the flower.”). Since the semantic interference effect is often interpreted as a reflection of difficulty in lexical selection [20], these results were considered as evidence for clause to be the scope of lexical planning. One way to explain this discrepancy is that the picture pairs in Meyer [37] were presented on the screen for only 800 ms, shorter than the mean response latencies observed in her study. It means that in many trials, the picture pairs disappeared before participants started articulation. While in current study the picture pairs were presented on the screen for 4,000 ms, much longer than the mean response latencies. The manipulations in Meyer [37] might force participants paying more attention to the lexical retrieval and attempting to encode the second noun more thoroughly, in order to comply with the experimental requirement of fast and accurate responses [15].

On the contrary, was the radically incremental scope observed in the current study an artifact caused by the long stimulus presentation? The answer is no, at least compared to the evidence for functional phrase hypothesis [13, 15]. It is known that the lexical planning scope is flexible for many reasons, such as time pressure [22], variable utterance formats [23], and the availability of the words and structures [4, 14]. Firstly, the duration of stimulus presentation in current study was similar to those studies which found evidence in support of the functional phrase hypothesis. Moreover, the nouns and sentence structures were repeated circularly in current study, which make the lexical planning scope tend to be larger [4, 14, 23]. Thus the conclusion of current study should not be attributed to the long stimulus presentation in the experiment.

Another question is whether the radically incremental scope observed in the current study an artifact caused by formulaic production. Schnur [38] proposed that the repetition of utterance format across trials may narrow down the phonological planning scope. However, this seems not to be the case in the situation here. As mentioned above, Meyer [37] also used formulaic production and concluded a much larger scope of lexical planning (the initial clause). On the contrary, Zhao and Yang [19] and Schriefers, Teruel and Meinhausen [39] used variable utterances in the picture-word interference experiments and found the semantic interference effect on the first content word (noun or verb) only. Thus the radically incremental scope of lexical planning observed in the current study is not likely an artifact caused by formulaic production.

This conclusion seems to contradict findings from other relevant studies, such as studies on horizontal information flow in sentence production [5, 40]. For example, Yang and Yang [40] tested the semantic information flow by asking participants to produce sentence with the structure “the N1 and the N2 are above/below/on the left/right of the N3”, while manipulating the semantic relations between the N1 and the N2. They found that onset latencies were longer when N1 and N2 were semantically related than unrelated. Thus they concluded that the second noun had been not only semantically accessed but also interacted with the retrieval of the first noun. However, if lexical planning is radically incremental, the second noun would not be lexically retrieved, thus there would be no information flow, or at least no influence of the latter words on the former words (i.e., no influence by the N2 on the N1). How to explain the discrepancy among these studies? Notice that in Yang and Yang’s study the N1 and the N3 were the names of presented pictures while the N2 was a printed word, which is quite different from several pictures aligned to produce sentences as in Griffin [10] and the current study. As we know, the printed word has a privileged access compared to the picture name [21]. Thus it is reasonable to speculate that even though only the first noun (N1) is lexically selected before speech onset, the N2 (printed word) may possibly be activated to some extent, and interfere with the retrieval of the N1.

The findings from some previous studies are also not readily compatible with radically incremental production. Schnur, Costa, and Caramazza [41] found that the semantic interference effect could extend to the intransitive verb in the sentence production such as “She jumps”. However, the effect of semantic interference on verbs is often unstable, even in the bare word naming task [39, 41]. Moreover, as a verb could be conceptually or grammatically important independent of its status as a lexical head of phrase [42] and results found for verb may not easily generalize to other content words, such as nouns. Schnur [38] concluded that the phonological planning before articulation is for the whole phonological phrase. However, is worth noticing that in Schnur’s study [38] the picture disappeared at speech onset, which may lead the participants to adopt large planning scope to achieve the experiment goal, as discussed previously with Meyer’s [37].

Another point we should notice is that the procedure used in the current study involved repetitions, which is the typical manipulation in the semantic blocking paradigm, but different from previous studies. Consequently, an important question for future studies, therefore, will be to test the radically incremental scope of lexical planning in more natural conditions with fewer repetitions and less formulaic constructions.

In the current study, we obtained ERP recordings during the speech production task. The cortical network involved in lexical production is widely distributed and predominantly located in the left hemisphere, basing on the functional neuroimaging data. The most influential spatio-temporal model of lexical production has been proposed by Indefrey and Levelt [43] (see a new version in [44]). This model is based on a cognitive processing model [20], which includes three processing stages: conceptual preparation (100–200 ms post picture onset), lexical selection (175–250 ms on average post picture onset), and form encoding (217–530 ms). The ERP effect of semantic blocking observed in the current study is consistent with previous results [29–30, 32] and the model of Indefrey and Levelt [43] in terms of its time window. However, the polarity and scalp distribution of this ERP effect is controversy in the literature. The results of Maess et al. [30] and Aristei et al. [29] showed a larger negative wave for the homogeneous compared to the heterogeneous condition over temporal regions, while Janssen et al. [32] obtained a reversed ERP effect pronounced at anterior electrodes. The polarity and scalp distribution of the semantic blocking effect in the current study are similar to the ones of Janssen et al. [32]. As Janssen et al. [32] claimed, the results of Maess et al. [30] and Aristei et al. [29] were obtained using post-hoc analyses involving only a subset of trials and participants, which might have biased their results. Moreover, the polarity of the effect observed in the current study and Janssen et al. [32] is comparable to Jescheniak et al.’s [45–46] studies with the picture–word interference task. It was observed that the ERP waveform induced by the target word was less negative when the picture name and the distractor word were semantically related than unrelated control condition in the time window of 250–400 ms [45]. Thus, our results provide further evidence for the semantic blocking effect in ERPs, and demonstrate that this effect can be used in speech production studies for longer utterances, such as sentences. However, we need to pay attention on the limitations of the ERPs in studying the long utterance production. Firstly, as the ERP data is very sensitive to the movement, it is only valid in the planning period, which limits the questions studied by the ERPs. Besides, there are many processes involved in the planning period, which needs strict control of the design. For example, in the current study the sentence structure needs to be constant in each block so that the semantic effect in ERPs is not overwhelmed. Even so, this study provides a valid case of studying the long utterance production with ERPs, and so will help inform future research in this area.

Another finding of the current study is the effect of sentence type observed both in Experiments 1 and 2. That is, CNP utterances need more time to prepare compared to the PP utterances, and the ERP effect of sentence type was consistently observed at anterior and central electrodes in the time window of 100–150ms and 300–400ms. Considering the absence of the semantic blocking effect of the second noun in Experiment 2, the difference in onset latencies between CNP and PP structures is unlikely to reflect lexical planning scope. Moreover, the effect of sentence type in ERPs seems to reflect syntactic and/or visual factors. According to a visual inspection, the ERPs in the time window of 100–150ms revealed a P1-N1 complex which is typically associated with the presentation of visual stimuli. It is speculated that the ERPs in the time window of 300–400ms may reflect the syntactic processing of the sentence structure, but this needs further investigation. Based on these results, it is not clear yet in which time window the ERP effect is associated with the latency effect and which processing stage it should be attributed to, but visual and syntactic processing seem to be reasonable factors. Although Zhao et al. [15] had tested the potential influence of the syntactic and visual factors on the latency effect between CNP and PP utterances, their results did not rule out these influences definitively. They indeed found a trend for a visual effect, and the potential attribution of structural planning to the latency effect could not be excluded if both the CNP and PP structures were encoded during the previewing period.

As discussed in relation to Experiment 2, if the effect in the time window of 300–400ms reflected the syntactic processing of the sentence structures, considering the semantic blocking effect in 250–350ms time window at the same time, these results may suggest that the first content word is processed slightly prior to the processing of syntactic structures. This is in line with an incremental account of production. There are two major views of incremental hypotheses. The radically incremental view suggests that the conceptually activated words would drive the selection of syntactic structures, while the relatively weak incremental view proposes that the highly activated words may influence the selection of alternative syntactic structures [47]. We do not aim to differentiate these two views here, as there was no choice between different sentence structures in the current study. Moreover, our speculation that there may be an ERP effect for the planning of sentence structure will needs further consideration, as the effect in the time window of 300–400ms in which we observed this possible effect of syntactic planning may reflect other processes related to the visual factors [36]. However, this speculation is in line with the previous findings of incremental production model, and may inspire further ERP study to test these views.

It was also notable that we did not find any evidence, in onset latencies or ERPs, for the phrasal unit as the lexical planning scope. This does not mean that the lexical planning scope cannot be the first noun phrase. Our conclusion may be suitable only in the conditions of the current study. Even in the same conditions, semantic effects of the second noun may be observed in other replications. However, we indeed did a replication in the Experiment 2 (the Session 2) and found no evidence for phrasal planning scope. On the other hand, according to previous studies [4, 14, 23] the lexical planning scope tends to be larger in circumstances with highly available words and syntactic structures, as in the present study. Thus the absence of any evidence for phrasal planning scope in the current study should be considered tenable. The semantic blocking effect of the second noun in ERPs may also be observed in late time windows such as later than 600ms post stimulus. We analyzed the data in ERPs in the time window of 600–1000ms, but found no sign of semantic blocking effect of the second noun. However, as the trials in which speakers began their articulation during or before this time window had to be excluded from the analysis of ERPs, only a small number of trials were left and this may have affected these analyses.

To conclude, the results of our study provide new evidence for the radically incremental hypothesis of lexical planning scope. With the fine time resolution of the ERP technique, the current study showed no indication of the second noun being lexically processed before the speech onset, although larger lexical scope has been proposed by previous studies. Thus when the speakers had enough time and information to prepare the coming utterances, and when the utterance structure and the elements within the utterance are highly available, they would lexically select words one by one. This study also provides an example of studying the long utterance production with ERPs, which is likely to inspire further applications of the ERP technique in this area.

## Supporting Information

S1 Appendix Stimuli used and their arrangement in Experiment 1. (DOC)

S2 Appendix The arrangement of the items in Experiment 2. (DOC)

S1 File Data used for analysis of onset latencies in Experiments 1 and 2. (XLS)

S2 File Data used for average waveform of ERP in CNP_Homogeneous condition in Experiment 1. (AVG)

S3 File Data used for average waveform of ERP in CNP_Heterogeneous condition in Experiment 1. (AVG)

S4 File Data used for average waveform of ERP in PP_Homogeneous condition in Experiment 1. (AVG)

S5 File Data used for average waveform of ERP in PP_Heterogeneous condition in Experiment 1. (AVG)

S6 File Data used for average waveform of ERP in CNP_Homogeneous condition in Experiment 2_Session 1. (AVG)

S7 File Data used for average waveform of ERP in CNP_Heterogeneous condition in Experiment 2_Session 1. (AVG)

S8 File Data used for average waveform of ERP in PP_Homogeneous condition in Experiment 2_Session 1. (AVG)

S9 File Data used for average waveform of ERP in PP_Heterogeneous condition in Experiment 2_Session 1. (AVG)

S10 File Data used for average waveform of ERP in CNP_Homogeneous condition in Experiment 2_Session 2. (AVG)

S11 File Data used for average waveform of ERP in CNP_Heterogeneous condition in Experiment 2_Session 2. (AVG)

S12 File Data used for average waveform of ERP in PP_Homogeneous condition in Experiment 2_Session 2. (AVG)

S13 File Data used for average waveform of ERP in PP_Heterogeneous condition in Experiment 2_Session 2. (AVG)
