# Supplementary material for: Lexical Planning in Sentence Production Is Highly Incremental: Evidence from ERPs
Source: PLoS One. 2016 Jan 5;11(1):e0146359. doi: 10.1371/journal.pone.0146359 (PMC4701458; doi:10.1371/journal.pone.0146359)
Supplement: S1 Appendix — (DOC) [file pone.0146359.s001.doc]

**S1 Appendix**

Appendix A.1. Stimuli used in Experiment (The corresponding Chinese pronunciation was presented in slices)

| Category | Items | | |
| --- | --- | --- | --- |
| Zoo animals | gorilla /xing’xing/ | elephant /da’xiang/ | zebra /ban’ma/ |
| Fruits | apple /ping’guo/ | banana /xiang’jiao/ | grapes /pu’tao/ |
| Furniture | dresser /chou’ti/ | chair /yi’zi/ | couch /sha’fa/ |
|  |  |  |  |
| Transports | airplane /fei’ji/ | car /qi’che/ | boat /chuan/ |
| Musical instruments | drum /gu/ | guitar /ji’ta/ | piano /gang’qin/ |
| Body parts | ear /er’duo/ | finger /shou’zhi/ | eye /yan’jing/ |

Appendix A.2. The arrangement of the items in Experiment 1

|  | Block1 | | Block2 | | Block3 | |
| --- | --- | --- | --- | --- | --- | --- |
| Position | N1 | N2 | N1 | N2 | N1 | N2 |
| Homogeneous | gorilla | boat | apple | airplane | dresser | car |
|  | elephant | drum | grapes | guitar | couch | piano |
|  | zebra | finger | banana | eye | chair | ear |
| Heterogeneous | elephant | airplane | gorilla | car | zebra | boat |
|  | apple | piano | grapes | drum | banana | guitar |
|  | dresser | finger | couch | eye | chair | ear |
