# Supplementary material for: Lexical Planning in Sentence Production Is Highly Incremental: Evidence from ERPs
Source: PLoS One. 2016 Jan 5;11(1):e0146359. doi: 10.1371/journal.pone.0146359 (PMC4701458; doi:10.1371/journal.pone.0146359)
Supplement: S2 Appendix — (DOC) [file pone.0146359.s002.doc]

**S2 Appendix**

Appendix B.1. The arrangement of the items in Experiment 2_Session 1

|  | Block1 | | Block2 | | Block3 | |
| --- | --- | --- | --- | --- | --- | --- |
| Position | N1 | N2 | N1 | N2 | N1 | N2 |
| Homogeneous | boat | gorilla | airplane | apple | car | dresser |
|  | drum | elephant | guitar | grapes | piano | couch |
|  | finger | zebra | eye | banana | ear | chair |
| Heterogeneous | airplane | elephant | car | gorilla | boat | zebra |
|  | piano | apple | drum | grapes | guitar | banana |
|  | finger | dresser | eye | couch | ear | chair |

Appendix B.2. The arrangement of the items in Experiment 2_Session 2

|  | Block1 | | Block2 | | Block3 | |
| --- | --- | --- | --- | --- | --- | --- |
| Position | N1 | N2 | N1 | N2 | N1 | N2 |
| Homogeneous | gorilla | airplane | elephant | drum | zebra | eye |
|  | grapes | car | banana | guitar | apple | finger |
|  | dresser | boat | couch | piano | chair | ear |
| Heterogeneous | zebra | airplane | elephant | car | gorilla | boat |
|  | banana | drum | grapes | piano | apple | guitar |
|  | dresser | eye | chair | ear | couch | finger |
